# Coronary plaque burden assessed by coronary CT angiography in individuals with type 1 diabetes and healthy controls

**DOI:** 10.1007/s10554-026-03640-w

**Published:** 2026-02-13

**Authors:** Cheyenne Y. Y. Beverloo, Shirin Ibrahim, Nick S. Nurmohamed, Coco M. Fuhri Snethlage, Mijra Koning, Andrew J. Murphy, Erik H. Serné, R. Nils Planken, Max Nieuwdorp, Erik S. G. Stroes, Nordin M.J. Hanssen

**Affiliations:** 1 Department of Vascular Medicine, Amsterdam UMC, University of Amsterdam, Amsterdam, The Netherlands; 2 Department of Cardiology, Amsterdam UMC, Vrije Universiteit Amsterdam, Amsterdam, The Netherlands; 3 Hematopoiesis and Leukocyte Biology, Baker Heart and Diabetes Institute, Melbourne, Australia; 4 Diabeter Centrum Amsterdam, Amsterdam UMC, Amsterdam, The Netherlands; 5 Department of Radiology and Nuclear Medicine, Amsterdam UMC, University of Amsterdam, Amsterdam, The Netherlands

**Keywords:** Coronary artery disease, Type 1 diabetes, Coronary CT angiography, Atherosclerosis imaging

## Abstract

Cardiovascular disease (CVD) remains a prominent cause of death in individuals with type 1 diabetes (T1D). It is unknown whether guideline-recommended initiation of statin therapy at the age of 40 years is adequate for primary prevention of CVD. We evaluated the coronary plaque burden in asymptomatic individuals with T1D compared with healthy controls. Individuals with T1D who used continuous glucose monitoring (CGM) sensors were compared with healthy controls. CGM data from ≥ 1 year prior to enrollment were obtained. All participants underwent coronary computed tomography angiography, which was followed by atherosclerosis imaging-quantitative computed tomography. Fifty-eight individuals with T1D and 45 healthy controls (mean age 42.1 ± 4.9 vs. 41.0 ± 3.5 years) participated. The participants with diabetes had a median disease duration of 24 years [IQR 17, 30]. The prevalence of significant coronary plaque was similar in both groups (31% vs. 22%) (OR 2.71 [95% CI 0.9 to 9.07]), as were plaque volumes and the presence of high-risk plaque, even after adjusting for clinical risk factors. Participants with T1D had a median time in range of 71% [IQR 52, 79] and a mean HbA1c of 55 ± 12 mmol/mol. The CGM metrics (time in range, time above range, time below range and glucose coefficient of variation) were not associated with plaque volumes. Individuals with long-standing well-regulated T1D and on-target lipid levels and blood pressure, exhibited a similar coronary plaque burden compared with healthy controls. These findings support early and aggressive risk factor management in T1D.

**Graphical abstract:**

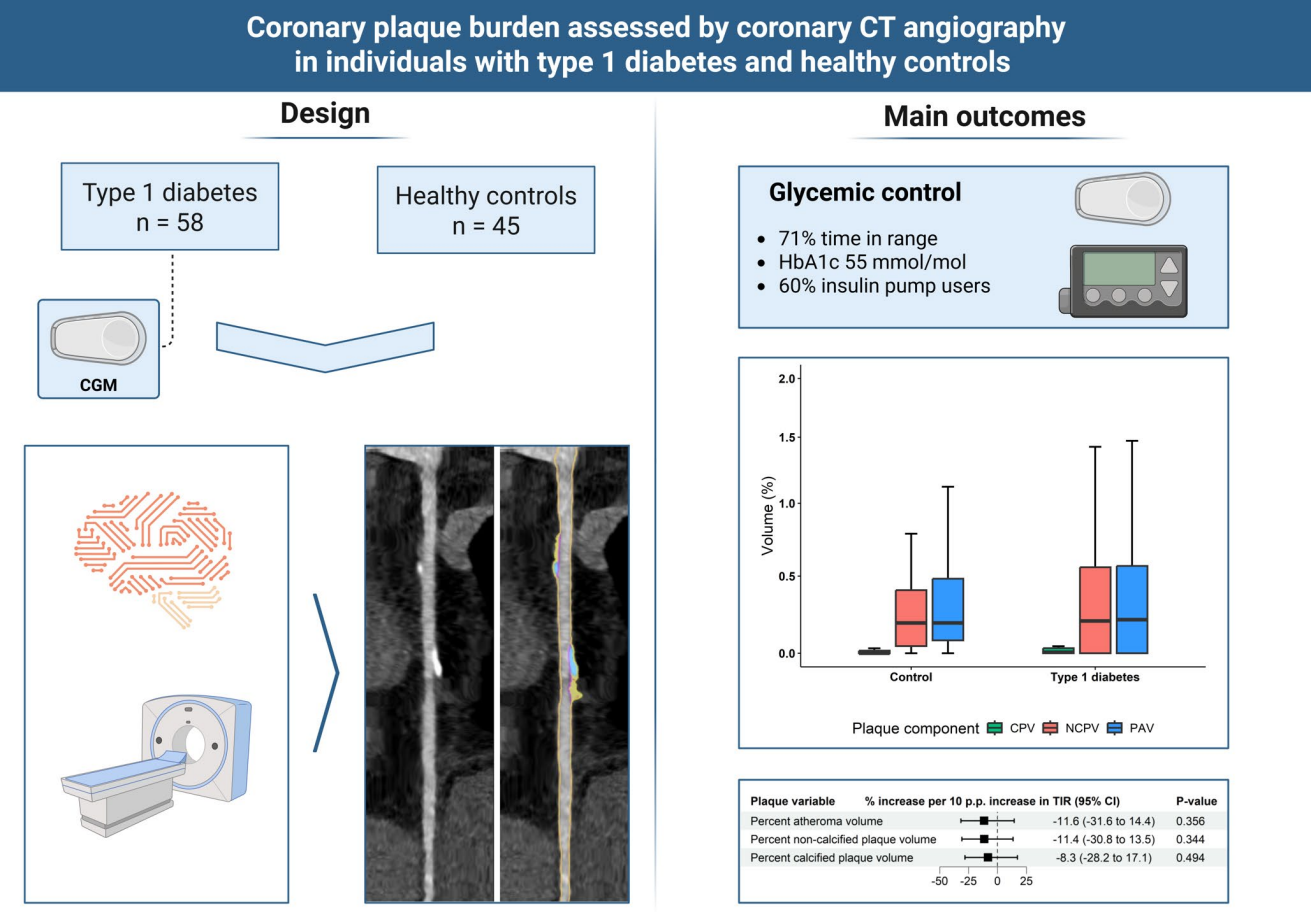

**Supplementary Information:**

The online version contains supplementary material available at 10.1007/s10554-026-03640-w.

## Introduction

Despite significant advancements in diabetes care over the recent decades, the global life expectancy of individuals with type 1 diabetes remains shortened up to 24 years compared with the general population [1]. In fact, there is a well-established association between type 1 diabetes and cardiovascular disease (CVD), with cardiovascular complications representing the leading cause of death in adults with type 1 diabetes [2–4]. Despite these impressive numbers, cardiovascular risk management in individuals with type 1 diabetes has lagged behind that of individuals with type 2 diabetes.

The 2023 European Society of Cardiology and the 2024 American Diabetes Association (ADA) guidelines recommend initiating statin therapy in individuals with type 1 diabetes above the age of 40 years, irrespective of low-density lipoprotein cholesterol (LDL-C) levels [5, 6]. Recently, we demonstrated that individuals with familial hypercholesterolaemia (FH) individuals indeed exhibit a higher coronary plaque burden at the age of 40 years when compared with normolipidemic healthy controls [7]. As individuals with type 1 diabetes also have an increased lifetime CVD risk, it therefore raises the question of whether initiating lipid-lowering therapy (LLT) at an age of 40 years may be too late to prevent atherosclerotic plaque formation. Previous studies evaluated coronary plaque formation in type 1 diabetes using coronary computed tomography angiography (CCTA), focusing on older [8, 9], or very young individuals [10]. The coronary plaque burden in middle-aged individuals with type 1 diabetes remains unexplored.

The implementation of technological devices in diabetes management, such as insulin pumps and continuous glucose monitoring (CGM) sensors, have markedly improved glycemic control [11–14]. It has been observed that the use of insulin pumps is associated with a lower cardiovascular mortality compared with multiple daily insulin injections [12, 15]. Poor glycemic control has been shown to contribute to the increased CVD-rate in individuals with type 1 diabetes [16, 17]. CGM metrics provide a better indication of glycemic control and variability, yet the association between CGM metrics and CVD has not been investigated in type 1 diabetes.

This study aimed to evaluate the coronary plaque burden and plaque characteristics in middle-aged individuals with long-standing type 1 diabetes compared with age- and sex-matched healthy controls, using CCTA followed by atherosclerosis imaging-quantitative computed tomography (AI-QCT). Within the diabetes cohort, we also evaluated the association between CGM metrics, and coronary plaque burden and plaque characteristics.

## Materials and methods

This observational study included a cohort of individuals with type 1 diabetes who were using CGM sensors for at least two years. These participants were matched to a cohort of healthy controls based on age and sex. Participants were recruited from Amsterdam and surrounding area and from the outpatient clinic of Amsterdam University Medical Centers (Amsterdam, the Netherlands). Participants aged between 35 and 55 years with a type 1 diabetes diagnosis of at least 5 years, were enrolled between October 2023 and March 2024. Participants were excluded if they had previously used LLT, or if they had a history of renal insufficiency, atrial fibrillation, or CVD (transient ischemic attack, cerebrovascular accident, angina, or myocardial infarction). The control group consisted of healthy volunteers.

This study was approved by the Institutional Review Board of Amsterdam UMC (reference number 2023.0649), and was conducted in accordance with the Declaration of Helsinki. All participants provided written informed consent.

### Study procedures and CGM metrics

Participants completed questionnaires regarding diabetes-related characteristics, their general health and lifestyle, medication use and family history. CGM data of at least one year prior to the study visit were collected. All participants used CGM sensors manufactured by Abbott (FreeStyle Libre 2 or 3), Medtronic (Enlite or Guardian) or Dexcom (G6). Average glucose, time in range (TIR), time above range (TAR), time below range (TBR), and glucose coefficient of variation (CV) were calculated by the algorithm of the CGM provider. These variables were obtained from the reports. Following the ADA recommendations for glycemic targets, the glucose ranges for TIR, TAR and TBR were set at 3.9–10.0 mmol/L, > 10.0 mmol/L, and < 3.9 mmol/L, respectively [18]. Additionally, the number of hypoglycemic events and average duration of hypoglycemic events were obtained. In participants with Medtronic or Dexcom sensors, the number of hypoglycemic events were manually read and their average duration was manually calculated. A hypoglycemic event was defined as a glucose concentration < 3.9 mmol/L with a duration longer than 15 minutes, in accordance with the definition Abbott maintains. Of all obtained CGM metrics, the weighted average was calculated over the available time period.

### CCTA acquisition and AI-QCT analysis

All participants with type 1 diabetes underwent combined coronary artery calcium (CAC) scoring and CCTA using a third-generation 2 × 192 slice dual source CT scanner (SOMATOM Force, Siemens Healthineers, Germany). Prior to the CCTA, participants were orally administered metoprolol if their heart rate exceeded 65 beats per minute and all participants were administered 400 mcg of sublingual nitroglycerin. CAC scores were obtained using a high-pitch helical (Flash) scan mode. CCTA images were acquired with automated tube voltage and tube current modulation (CAREKv, CAREDose 4D, Siemens Healthineers, Germany), using a prospective ECG-triggered protocol at 70% of the R-R interval. During the CCTA, patients received iodine contrast fluid intravenously (Xenetix 350, Guerbet Nederland B.V., Gorinchem, the Netherlands). A weight and kV dependent contrast dose was used following a test bolus.

**Fig. 1:**
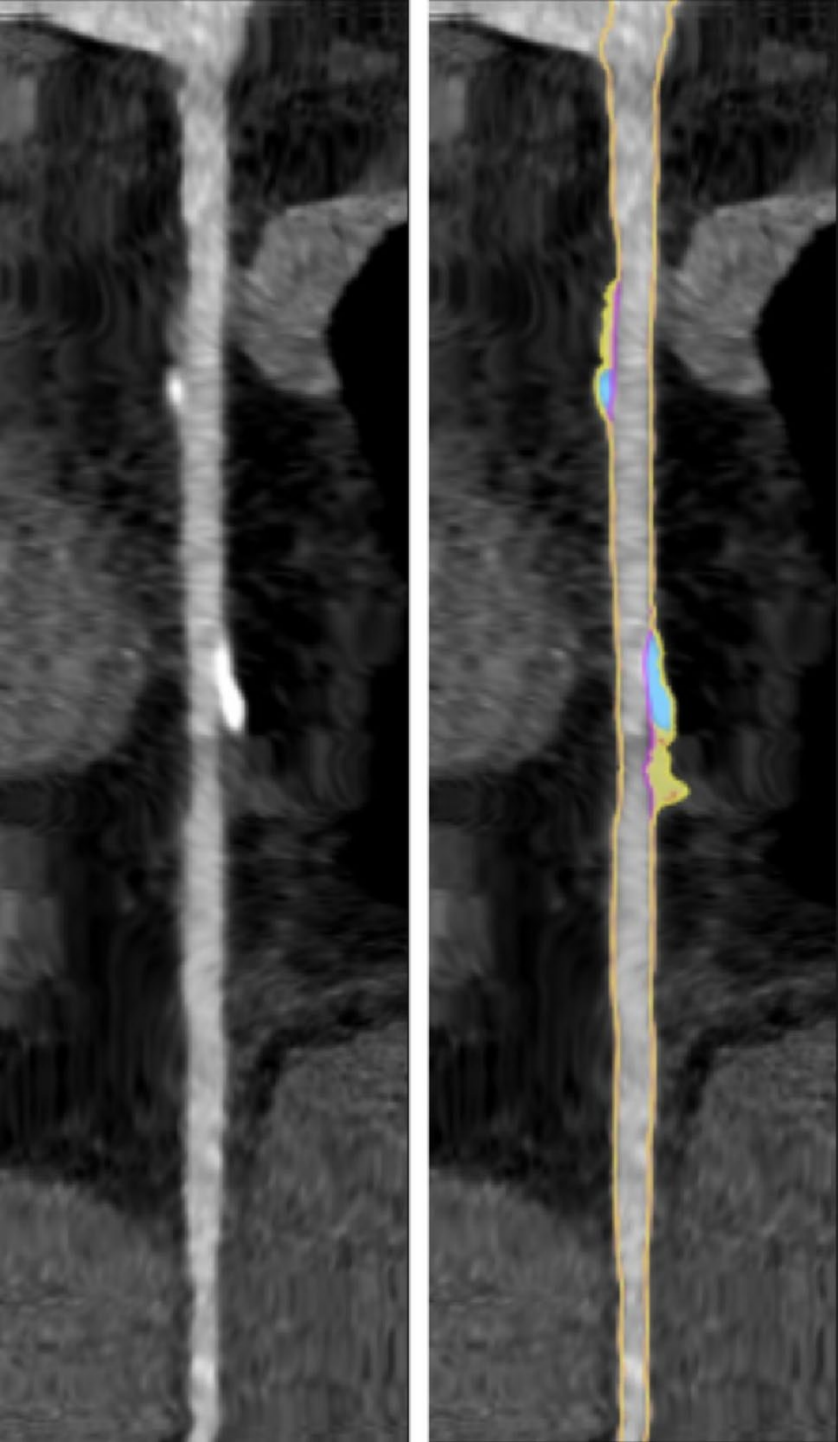
AI-QCT analysis of a female 51 year-old participant with a diabetes duration of 32 years. Shown are straightened reformatted CCTA images of the right coronary artery). On the right-hand image, AI-QCT additions show lumen boundaries (yellow lines), lumen-plaque borders (pink lines), non-calcified plaque (yellow area), calcified plaque (blue area)

CCTA images were analyzed by Food and Drug Administration (FDA)-cleared automated AI-QCT software (Cleerly Inc., Denver, CO; Fig. 1). This software service uses a series of validated convolutional neural networks for image quality assessment, coronary segmentation and labeling, lumen wall evaluation, vessel contour determination, and plaque characterization. The validation of AI-QCT in multicenter trials has been described previously [7, 19–25].

Coronary segments with a diameter ≥ 1.5 mm were included in the analysis. If impaired image quality was present due to motion, poor opacification, beam hardening, or other artifact, the part of the coronary artery with poor quality was excluded from the analysis. The coronary arteries were evaluated for the presence of coronary atherosclerosis, defined as any tissue structure > 1 mm^2^ within the coronary artery wall that was differentiated from the surrounding epicardial tissue, epicardial fat and the vessel lumen itself. Coronary lesions were summated to calculate the total plaque volume (mm^3^). Plaque volumes were categorized based on the Hounsfield Unit (HU) attenuation, with low-density non-calcified plaque volume defined as a HU range from 0.1 and < 30, non-calcified plaque volume as a HU range between − 30 and + 350, and calcified plaque volume defined as HU > 350 [26]. Lastly, to account for variation in coronary artery volume, coronary plaque volume was normalized to vessel volume, calculated as plaque volume divided by vessel volume x 100%. These normalized volumes were used to calculate percent atheroma volume, percent non-calcified plaque volume and percent calcified plaque volume. Significant presence of coronary plaque was defined as a percent atheroma volume above or equal to 0.5% (~ 10 mm^3^) [7]. Presence of low-density non-calcified plaque was defined as any presence of low-density non-calcified plaque and high-risk plaque was defined as the combined presence of positive remodeling (remodeling index > 1.1) and low-density non-calcified plaque in one lesion.

### Statistical analysis

Differences in coronary plaque burden and plaque characteristics were compared between participants with type 1 diabetes and controls. For comparing continuous variables (percent atheroma volume, non-calcified plaque volume, and calcified plaque volume) a Wilcoxon rank-sum test was used and for categorical variables (presence of high-risk plaque and presence of low-density non-calcified plaque) a chi-square test. The association between CGM metrics (TIR, TAR, TBR, and glucose CV) and the different plaque volumes was assessed using multivariable linear regression models, adjusted for age, sex, systolic blood pressure, body mass index [BMI], LDL-C, triglycerides, smoking history, disease duration, presence of diabetic retinopathy, and family history of CVD. Due to the right-skewed distribution of TBR, this variable was log_2_-transformed. Similarly, percent atheroma volume, non-calcified plaque, volume and calcified plaque volume were log_2_-transformed. CGM data was considered insufficient if less than a year of CGM data was available, or if the average CGM sensor activation time did not exceed 70%. Consequently, these participants were excluded from CGM analyses. Additionally, an estimation of the cumulative HbA1c exposure was calculated using a minimum of one historical HbA1c measurement, at least one year prior to inclusion. The cumulative HbA1c exposure was measured as the area under the curve, using the trapezoidal rule method. The association between cumulative HbA1c exposure and the plaque volumes was assessed similar to the assessment of CGM metrics and the plaque volumes.

Data are reported as numbers (%) for categorical variables, mean ± SD for normally distributed variables, and median [IQR] for non-normally distributed variables. Two-sided P values < 0.05 were considered significant. RStudio version 4.3.2 was used to perform all statistical analyses.

## Results

### Study population

A total of 58 participants with type 1 diabetes and 45 healthy controls (mean age 42.1 ± 4.9 years vs. 41.0 ± 3.5 years; 33% vs. 38% male, respectively) were included in the present study (Table 1**)**. Systolic blood pressure measured during the study visits was 122 ± 15 mmHg in the diabetes group vs. 115 ± 13 mmHg in the control group (*p* = 0.018). The participants with diabetes had a significantly lower LDL-C compared with the controls (2.60 ± 0.91 mmol/L vs. 3.00 ± 0.84 mmol/L; *p* = 0.004), while high-density lipoprotein cholesterol was higher in the participants with diabetes (1.84 ± 0.55 mmol/L vs. 1.53 ± 0.45 mmol/L; *p* = 0.005). The participants with diabetes had a median disease duration of 24 years [IQR 17, 30], a mean HbA1c of 7.2 ± 3.2% (55 ± 12 mmol/mol), a median TIR of 71% [IQR 52, 79], a median TAR of 26% [IQR 17, 43], a median TBR of 2.2% [IQR 1.0, 4.8] and a mean glucose CV of 35 ± 7%.

**Table 1:** Participant characteristics

Characteristic	Type 1 diabetes *N* = 58	Controls *N* = 45	*p*-value
Age ± SD – years	42.1 ± 4.9	41.0 ± 3.5	0.471
Male sex – no. (%)	19 (33%)	17 (38%)	0.596
Body mass index ± SD – kg/m^2^	25.7 ± 4.5	25.8 ± 4.4	0.783
Active or previous smoking – no. (%)	23 (40%)	16 (36%)	0.671
Systolic blood pressure ± SD – mmHg	122 ± 15	115 ± 13	0.018
Diastolic blood pressure ± SD – mmHg	76 ± 9	76 ± 9	0.831
ACE inhibitor or ARB use – no. (%)	2 (3.4%)	1 (2.2%)	1.000
LDL cholesterol ± SD – mmol/L	2.60 ± 0.91	3.00 ± 0.84	0.004
HDL cholesterol ± SD – mmol/L	1.84 ± 0.55	1.53 ± 0.45	0.005
Triglycerides (IQR) – mmol/L	0.79 (0.61, 1.23)	0.86 (0.67, 1.48)	0.181
Lipoprotein(a) (IQR) – nmol/L	16 (8, 51)	17 (9, 65)	0.738
C-reactive protein (IQR) – mg/L	0.95 (0.60, 2.08)	0.90 (0.50, 1.50)	0.186
Age of diabetes onset (IQR) – years	18 (10, 26)	NA	NA
Disease duration (IQR) – years	24 (17, 30)	NA	NA
Diabetic retinopathy – no. (%)	21 (36%)	NA	NA
Proliferative retinopathy – no. (%)	4 (6.9%)	NA	NA
Diabetic neuropathy – no. (%)	2 (3.4%)	NA	NA
Urine microalbumin-creatinine ratio (IQR) – mg/mmol	0.32 (0.00, 0.58)	NA	NA
HbA1c ± SD – % (mmol/mol)	7.2 ± 3.2% (55 ± 12)	NA	NA
Cumulative HbA1c exposure ± SD – mmol/mol * years	1,310 ± 598	NA	NA
Insulin dose ± SD – units/day	44 ± 18	NA	NA
CGM sensor type – no. (%)		NA	NA
Freestyle Libre 2	26		
Freestyle Libre 3	3		
Medtronic Guardian	14		
Medtronic Enlite	1		
Dexcom G6	14		
Days of CGM data (IQR)	600 (488.810)	NA	NA
TIR (IQR) – %	71 (52, 79)	NA	NA
TAR (IQR) – %	26 (17, 46)	NA	NA
TBR (IQR) – %	2.2 (1.0, 4.8)	NA	NA
Glucose CV ± SD – %	35.9 ± 6.7	NA	NA
Hypoglycemic events (IQR) – no. per 90 days	34 (15, 74)	NA	NA
Severe hypoglycemia – no. of participants (%)	37 (63.8)	NA	NA
Insulin pump – no. (%)		NA	NA
None	23 (40%)		
Manual	14 (24%)		
Predictive low-glucose suspend	2 (3.4%)		
Hybrid closed-loop	15 (26%)		
DIY closed-loop	4 (6.9%)		

ACE, angiotensin-converting enzyme; ARB, angiotensin receptor blocker; LDL, low-density lipoprotein; HDL, high-density lipoprotein; TIR, time in rage; TAR, time above range; TBR, time below range; glucose CV, glucose coefficient of variation

### Coronary plaque burden: type 1 diabetes vs. controls

**Fig. 2:**
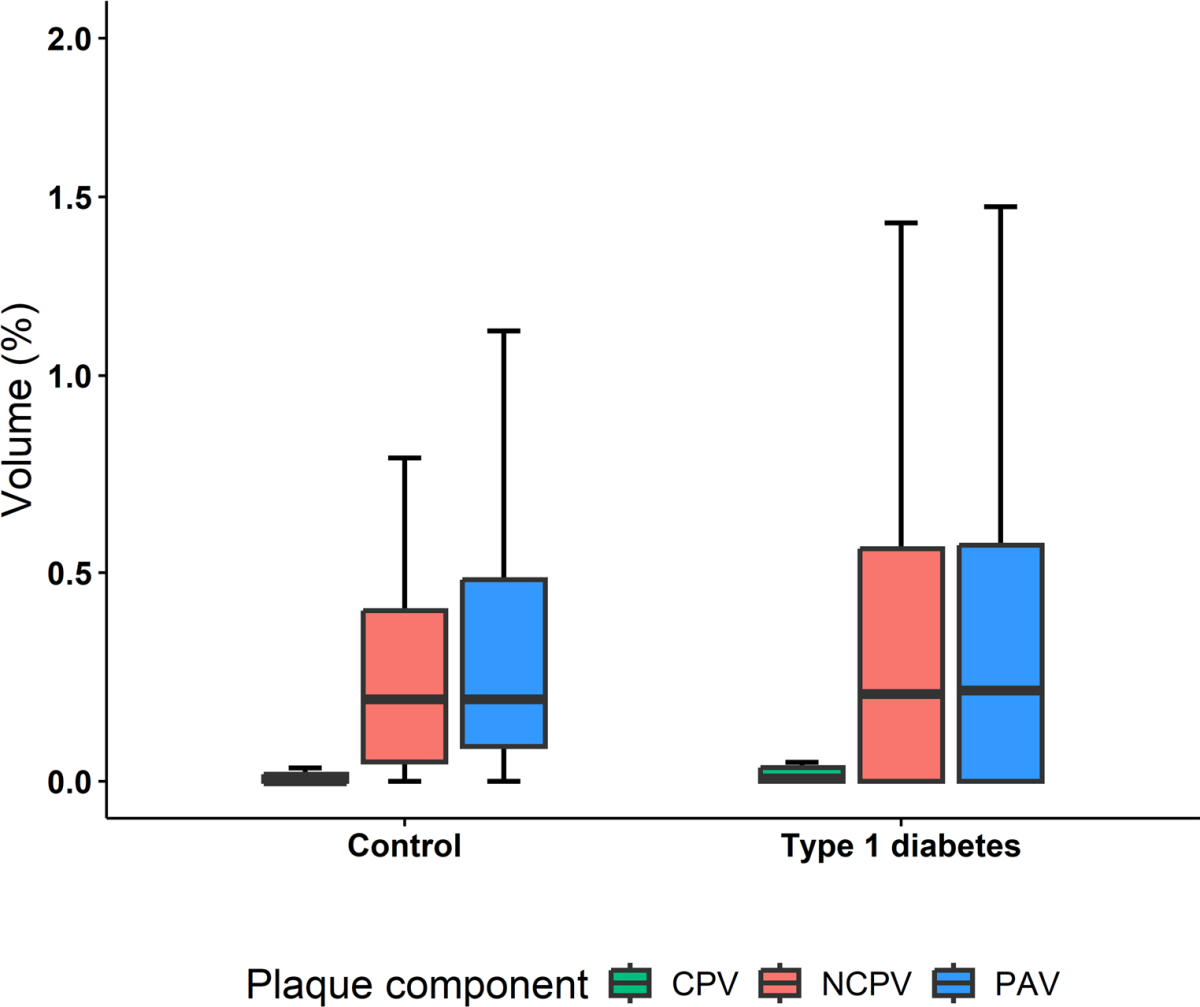
Boxplots of coronary plaque burden in healthy controls and type 1 diabetes participants. Shown are boxplots of the different plaque volumes for both the control group as well as the individuals with type 1 diabetes. CPV, percent calcified plaque volume; NCPV, percent non-calcified plaque volume; PAV, percent atheroma volume

The prevalence of significant presence of coronary plaque did not statistically differ between the participants with type 1 diabetes compared with the healthy controls (31% vs. 22%; adjusted OR 2.71 [95% CI 0.9 to 9.07]; *p* = 0.087; Table 2 and Supplementary Table S1). Furthermore, percent atheroma volume (0.22% [IQR 0.00, 0.57] vs. 0.19% [0.08, 0.48]; *p* = 0.848) and non-calcified plaque volume (0.21% [IQR 0.00, 0.56] vs. 0.19% [IQR 0.05, 0.41]; *p* = 0.720) were similar between both groups (Fig. 2). Both groups exhibited a low percentage of calcified plaque volume (0.01% [IQR 0.00, 0.03] vs. 0.00% [IQR 0.00, 0.02]; *p* = 0.033). Adjusted for traditional clinical risk factors (Supplementary Table S1), type 1 diabetes was not significantly associated with the total plaque volume (β = 0.18 [95% CI −1.81 to 2.16]; *p* = 0.863), percent atheroma volume (β = 0.34 [95% CI −0.54 to 1.21]; *p* = 0.455), non-calcified plaque volume (β = 0.36 [95% CI −0.47 to 1.2]; *p* = 0.397), or calcified plaque volume (β = 0.49 [95% CI −0.41 to 1.39]; *p* = 0.286). The prevalence of high-risk plaque was numerically higher in the type 1 diabetes group compared with the control group (17% vs. 6.7%; adjusted OR 4.86 [95% CI 0.79 to 53.98]; *p* = 0.124).

**Table 2:** Plaque burden type 1 diabetes participants vs. controls

Characteristic	Type 1 diabetes *N* = 58	Controls *N* = 45	*p*-value
Significant presence of coronary plaque – no. (%)	18 (31%)	10 (22%)	0.319
Total plaque volume (IQR) – mm^3^	6 (0, 13)	6 (2, 13)	0.812
Percent atheroma volume (IQR) – %	0.22 (0.00, 0.57)	0.19 (0.08, 0.48)	0.848
Percent non-calcified plaque volume (IQR) – %	0.21 (0.00, 0.56)	0.19 (0.05, 0.41)	0.720
Percent calcified plaque volume (IQR) – %	0.01 (0.00, 0.03)	0.00 (0.00, 0.02)	0.033
Presence of high-risk plaque	10 (17%)	3 (6.7%)	0.109
Presence of low-density plaque – no. (%)	11 (19%)	5 (11%)	0.275
Positive remodeling– no. (%)	19 (33%)	15 (33%)	0.951

### Association between CGM metrics and plaque burden

**Fig. 3:**
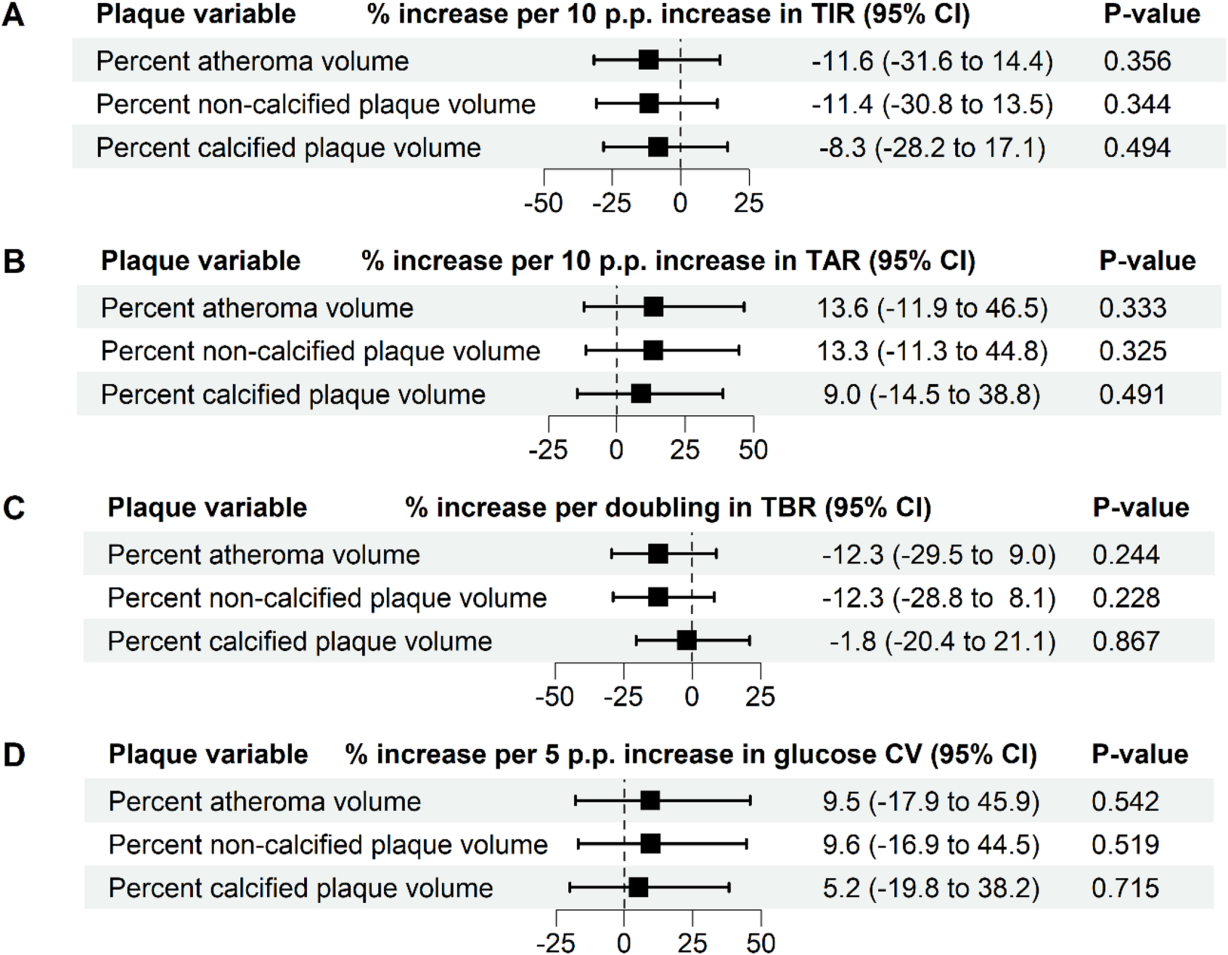
Associations between CGM-metrics and coronary plaque burden. Shown are the relative increases derived beta coefficients from linear regression models of time in range (**A**), time above range (**B**), time below range (**C**), and glucose CV (**D**) with the different plaque volumes (log_2_ transformed) as outcome variable. Models were adjusted for clinical risk factors (age, sex, systolic blood pressure, body mass index, LDL-C, triglycerides, smoking history, disease duration, presence of diabetic retinopathy, and family history of CVD). P.p, percentage point; TIR, time in rage; glucose CV, glucose coefficient of variation

**Fig. 4:**
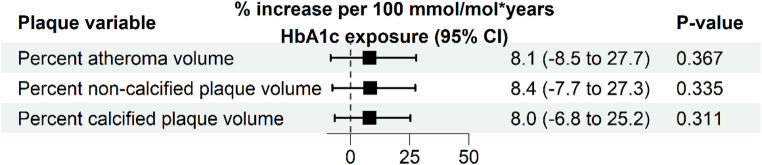
Associations between cumulative glycemic exposure (HbA1c mmol/mol*years) and coronary plaque burden in the CGM cohort. Shown are the relative increases derived beta coefficients from linear regression model of HbA1c with the different plaque volumes (log_2_ transformed) as outcome variable. Models were adjusted for clinical risk factors (age, sex, systolic blood pressure, body mass index, LDL-C, triglycerides, smoking history, disease duration, presence of diabetic retinopathy, and family history of CVD)

**Fig. 5:**
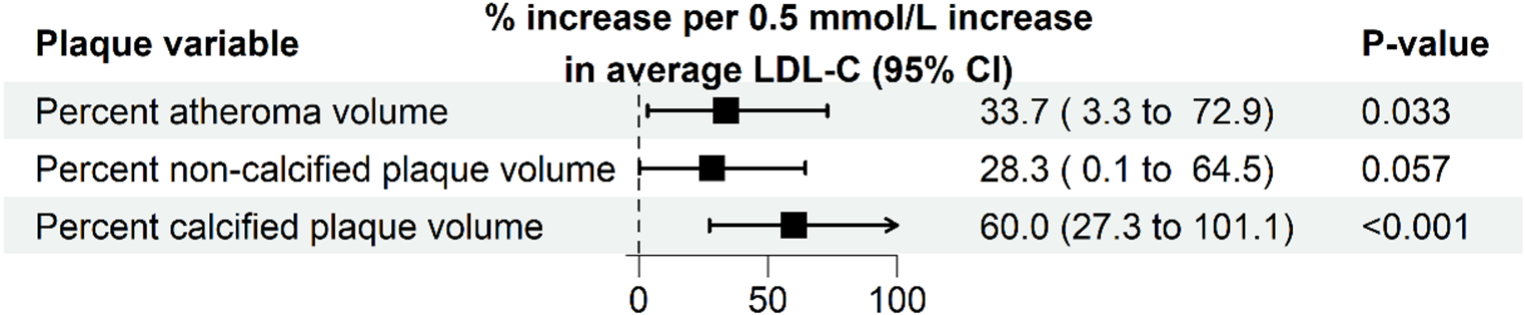
Associations between low-density lipoprotein cholesterol (LDL-C) and coronary plaque burden in the CGM cohort. Shown are the relative increases derived beta coefficients from linear regression model of LDL-C with the different plaque volumes (log_2_ transformed) as outcome variable. Models were adjusted for clinical risk factor (age, sex, systolic blood pressure, body mass index, triglycerides, smoking history, disease duration, presence of diabetic retinopathy, and family history of CVD)

Ten participants were excluded from the CGM analysis due to insufficient CGM data, resulting in 48 participants (CGM cohort). The characteristics of these remaining 48 participants, stratified by TIR above or below the median, are shown in Supplementary Table S2. The median number of days with available CGM data was 614 days (range 367–900 days [IQR 510–810 days]). The adjusted associations between TIR, TAR, TBR, and glucose CV in relation to the different plaque volumes are shown in Fig. 3. Adjusted for clinical risk factors, increases in TIR, TAR, TBR and glucose CV were not significantly associated with a relative change of percent atheroma volume, percent non-calcified plaque volume, or percent calcified plaque volume (all *p* > 0.05). Within the same cohort, the adjusted association between cumulative HbA1c exposure and the different plaque volumes was also not statistically significant (Fig. 4). In contrast, every 0.5 mmol/L increase in average LDL-C, adjusted for other clinical risk factors, was significantly associated with a relative increase of 33.7% of percent atheroma volume (95% CI 3.3 to 72.9; *p* = 0.033) and a 60.0% relative increase of percent calcified plaque volume (95% CI 27.3 to 101.1; *p* < 0.001) (Fig. 5). Lastly, the presence of diabetic retinopathy (Table 3) was significantly associated with percent atheroma volume (β = 1.31; 95% CI 0.16 to 2.46; *p* = 0.031), percent non-calcified plaque volume (β = 1.24; 95% CI 0.12 to 2.35; *p* = 0.034), and percent calcified plaque volume (β = 1.35; 95% CI 0.28 to 2.42; *p* = 0.017).

**Table 3:** Association between the presence of diabetic retinopathy and plaque burden

Plaque variable	β (95% CI)	*p*-value
Percent atheroma volume	1.31 (0.16 to 2.46)	0.031
Percent non-calcified plaque volume	1.24 (0.12 to 2.35)	0.034
Percent calcified plaque volume	1.35 (0.28 to 2.42)	0.017

Models were adjusted for age, sex, systolic blood pressure, BMI, smoking history, LDL-C, family history of CVD and as suggested by the reviewer earlier, triglycerides and heart rate

## Discussion

This study demonstrates that the coronary plaque burden in individuals with well-regulated, long-standing type 1 diabetes is similar to that of healthy controls. Furthermore, in this cohort of middle-aged individuals who did not receive LLT, CGM metrics were not associated with coronary plaque burden, in contrast to LDL-C levels, which exhibited a strong correlation with coronary plaque burden.

At a mean age of 42 years and with well-regulated glucose levels, the prevalence of significant coronary plaque on CCTA in participants with type 1 diabetes did not differ from healthy controls, which contrasts with the presence of marked atherosclerotic disease already present in individuals with FH at the age of 40 years [7]. This discrepancy highlights the concept that the atherogenicity of prolonged exposure to elevated LDL-C levels exceeds the potential atherogenic impact of elevated glucose levels. Nonetheless, our data contrasts with previous findings by Svanteson et al. [8, 9], who reported that 85% of the participants with type 1 diabetes exhibited presence of coronary plaque on CCTA, compared with 47% of the matched controls (*p* < 0.01). Notably, in their study, the participants with type 1 diabetes were markedly older (mean age 61.5 ± 7.1 years vs. 42.1 ± 4.9 years in the present study) with their diabetes duration being twice as long (median duration 51 years vs. median duration 24 years in the present study). In addition, the use of LLT or a history of CVD was not considered an exclusion criterion in the study by Svanteson, which likely resulted in a cohort with a more adverse cardiovascular risk profile. Another study by Madaj et al. found that 44% of the 25 participants with type 1 diabetes exhibited any coronary plaque on CCTA at a mean age of 26 ± 4 years [10], clearly exceeding the prevalence of coronary plaque in our study (31% at a mean age of 42 years). Most striking in the study by Madaj, was the poor glycemic control with a mean HbA1c of 9.0% (75 mmol/mol) vs. 7.2% (55 mmol/mol) in our study. Furthermore, they did not report on relevant characteristics, such as sex distribution, BMI, smoking status, and LDL-C levels.

Previous data has suggested that poor glycemic control is associated with cardiovascular events and mortality in type 1 diabetes [16, 27], while a large glycemic variability has been hypothesized as a potential atherogenic factor [28, 29]. We did not find an association between glycemic control parameters such as TIR, TAR, HbA1c, and glycemic variability and coronary plaque parameters. A major limitation of this finding relates to the fact that the participants with type 1 diabetes in our study were relatively well regulated, as indicated by the CGM metrics, HbA1c levels and the low prevalence of microvascular complications. Therefore, the present study lacked a poorly regulated comparator group. However, Lind et al. [16] suggested that even with a HbA1c below 7.0% (53 mmol/mol), individuals with type 1 diabetes remained at increased risk for cardiovascular mortality compared with controls, which increased as glycemic control worsened. A limitation of the study by Lind et al. is that their hazard ratio was not adjusted for other clinical risk factors, such as LDL-C. Our observations underscores that even at low levels, LDL-C is still correlated with coronary atherosclerosis. Furthermore, the study by Lind et al. was based on a cohort observed between 1998 and 2011, in contrast to the present study, which was conducted between 2023 and 2024. During this time span, significant improvements have been made in diabetes care with the introduction of insulin pumps and CGM devices, likely resulting in better management of cardiovascular risk factors.

Although the incidence of cardiovascular complications remains higher among individuals with type 1 diabetes than in healthy controls, the differences appear to have diminished between 1998 and 2014, a trend that may have been continuing during the last decade [4]. Rawshani et al. has shown that excess cardiovascular risk in type 1 diabetes is highly dependent on the co-occurrence of additional risk factors [30]. Our findings align with the results of Rawshani et al., demonstrating a 1.8 relative risk increase in incident myocardial infarction in the absence of risk factors, while this risk was increased up to 12.3 in the presence of risk factors. It could be hypothesized that the increasing availability of technologies such as insulin pumps and CGM sensors, combined with achievement of current targets of blood pressure, and lipid control may result in a lower plaque burden and a further decline in cardiovascular complications. The present is of major significance as we find in absence of major risk factors trends towards less plaque volume in type 1 diabetes, suggesting that well-managed type 1 diabetes may be less of a pro-atherogenic risk factor.

The current study has several limitations that require further discussion. First, the study was designed as a cross-sectional, single-center observational study with a limited sample size. Despite the adjustment for several potential confounders in multivariable models, some may be lacking. Second, the participants with type 1 diabetes enrolled in the current study are relatively healthy, which could be indicative of selection bias. The diabetes group had no major diabetes-related complications or other critical comorbidities and the disease was well-regulated, due to the assistance of their CGM sensors and the frequent use of insulin pumps. They had lower LDL-C levels compared with the control group, which is possibly caused by stringent cardiovascular risk management as part of the standard diabetes management. As the unaffected controls were healthy, they were less likely to be under strict surveillance for their cardiovascular health. Third, due to limitations in the CGM platforms, a maximum of 1.5 years of CGM data was available for the assessment of the association between glycemic control and coronary plaque burden in the majority of the participants, despite their prolonged use of the CGM device. To provide additional data on glycemic control, cumulative HbA1c exposure was calculated, with the use of historical measurements. Although this provides a good illustration of an individual’s trend regarding long-term glycemic control, it does not fully represent the true lifetime glycemic exposure. Next, HbA1c measurements were not available in the control group, which limits the potential for exploration of interactions between HbA1c levels and the groups. Furthermore, family history of CVD was high in the control group, as this group largely consisted of healthy family members of individuals with FH. This limits the use of this variable in the comparison between the two groups. Fourth, while the coronary plaque burden at a single time point is informative, it is not a hard clinical endpoint and not a direct marker of plaque progression or the risk of future cardiovascular events. Finally, the aforementioned risk of selection bias and residual confounding may have resulted in the comparable plaque burden in the diabetes group and the healthy controls. In the absence of significant differences, uncertainty remains. Future assessments with a longer follow-up should be performed to investigate whether individuals with well-regulated type 1 diabetes remain at a comparable coronary plaque burden to that observed in healthy controls later in life. Moreover, a population with more poorly regulated type 1 diabetes should be assessed to investigate whether CGM metrics are predictive of coronary atherosclerosis in this population.

In conclusion, this study demonstrates a comparable coronary plaque burden in individuals with well-regulated type 1 diabetes without CVD and healthy controls at an age of 42 years. Furthermore, within a group of individuals with adequate glycemic control, CGM metrics are not associated with the coronary plaque burden, contrary to LDL-C, which is a predictive indicator in this group. This suggests that our study was adequately powered to detect relevant associations between atherosclerotic risk factors and coronary plaque burden. However, further research is warranted to investigate whether the coronary plaque burden in individuals with type 1 diabetes remains comparable to that of healthy individuals later in life. Nonetheless, the present findings suggest that aggressive risk factor may result in a further decline in cardiovascular burden. These results therefore call for cautious optimism for the future and large-scale studies should reveal whether outcomes in type 1 diabetes will indeed improve further by focusing on improving glycemic control and risk factor modification.

## Supplementary Information

Below is the link to the electronic supplementary material.

Supplementary Material 1

## Data Availability

The datasets used and analyzed during the current study are available from the corresponding author upon reasonable request.

## References

[1] Gregory GA, Robinson TIG, Linklater SE, Wang F, Colagiuri S, de Beaufort C et al (2022) Global incidence, prevalence, and mortality of type 1 diabetes in 2021 with projection to 2040: a modelling study. Lancet Diabetes Endocrinol 10(10):741–60 36113507 10.1016/S2213-8587(22)00218-2

[2] Secrest AM, Becker DJ, Kelsey SF, Laporte RE, Orchard TJ (2010) Cause-specific mortality trends in a large population-based cohort with long-standing childhood-onset type 1 diabetes. Diabetes 59(12):3216–3222 20739685 10.2337/db10-0862 PMC2992785

[3] Gagnum V, Stene LC, Jenssen TG, Berteussen LM, Sandvik L, Joner G et al (2017) <article-title update="added">Causes of death in childhood‐onset type 1 diabetes: long‐term follow‐up. Diabet Med 34(1):56–63 26996105 10.1111/dme.13114

[4] Rawshani A, Rawshani A, Franzen S, Eliasson B, Svensson AM, Miftaraj M et al (2017) Mortality and cardiovascular disease in type 1 and type 2 diabetes. N Engl J Med 376(15):1407–18 28402770 10.1056/NEJMoa1608664

[5] Marx N, Federici M, Schutt K, Muller-Wieland D, Ajjan RA, Antunes MJ et al (2023) 2023 ESC guidelines for the management of cardiovascular disease in patients with diabetes. Eur Heart J 44(39):4043–4140 37622663 10.1093/eurheartj/ehad192

[6] American Diabetes A (2021) 10. Cardiovascular Disease and Risk Management: Standards of Medical Care in Diabetes-2021. Diabetes Care. ;44(Suppl 1):S125–S50 10.2337/dc21-S010 33298421

[7] Ibrahim S, Reeskamp LF, de Goeij JN, Hovingh GK, Planken RN, Bax WA et al (2024) Beyond early LDL cholesterol Lowering to prevent coronary atherosclerosis in Familial hypercholesterolaemia. Eur J Prev Cardiol 31(7):892–900 38243822 10.1093/eurjpc/zwae028

[8] Svanteson M, Holte KB, Haig Y, Klow NE, Berg TJ (2019) Coronary plaque characteristics and epicardial fat tissue in long term survivors of type 1 diabetes identified by coronary computed tomography angiography. Cardiovasc Diabetol 18(1):58 31054573 10.1186/s12933-019-0861-x PMC6500584

[9] Holte KB, Svanteson M, Hanssen KF, Haig Y, Solheim S, Berg TJ (2019) Undiagnosed coronary artery disease in long-term type 1 diabetes. The Dialong study. J Diabetes Complications 33(5):383–389 30846232 10.1016/j.jdiacomp.2019.01.006

[10] Madaj PM, Budoff MJ, Li D, Tayek JA, Karlsberg RP, Karpman HL (2012) Identification of noncalcified plaque in young persons with diabetes: an opportunity for early primary prevention of coronary artery disease identified with low-dose coronary computed tomographic angiography. Acad Radiol 19(7):889–893 22542200 10.1016/j.acra.2012.03.013 PMC4277701

[11] Kamrath C, Tittel SR, Kapellen TM, von dem Berge T, Heidtmann B, Nagl K et al (2021) Early versus delayed insulin pump therapy in children with newly diagnosed type 1 diabetes: results from the multicentre, prospective diabetes follow-up DPV registry. Lancet Child Adolesc Health 5(1):17–25 33253630 10.1016/S2352-4642(20)30339-4

[12] Derosa G, Catena G, Scelsi L, D’Angelo A, Raddino R, Cosentino E et al (2020) Glyco-metabolic control, inflammation markers, and cardiovascular outcomes in type 1 and type 2 diabetic patients on insulin pump or multiple daily injection (italico study). Diabetes Metab Res Rev 36(1):e3219 31642581 10.1002/dmrr.3219

[13] Rodbard D (2017) Continuous glucose monitoring: a review of recent studies demonstrating improved glycemic outcomes. Diabetes Technol Ther 19(S3):S25–S37 28585879 10.1089/dia.2017.0035 PMC5467105

[14] Beck RW, Riddlesworth T, Ruedy K, Ahmann A, Bergenstal R, Haller S et al (2017) Effect of continuous glucose monitoring on glycemic control in adults with type 1 diabetes using insulin injections: the DIAMOND randomized clinical trial. JAMA 317(4):371–8 28118453 10.1001/jama.2016.19975

[15] Steineck I, Cederholm J, Eliasson B, Rawshani A, Eeg-Olofsson K, Svensson AM et al (2015) Insulin pump therapy, multiple daily injections, and cardiovascular mortality in 18,168 people with type 1 diabetes: observational study. BMJ 350:h3234 26100640 10.1136/bmj.h3234 PMC4476263

[16] Lind M, Svensson AM, Kosiborod M, Gudbjornsdottir S, Pivodic A, Wedel H et al (2014) Glycemic control and excess mortality in type 1 diabetes. N Engl J Med 371(21):1972–82 25409370 10.1056/NEJMoa1408214

[17] Miller RG, Orchard TJ, Costacou T (2022) 30-year cardiovascular disease in type 1 diabetes: risk and risk factors differ by long-term patterns of glycemic control. Diabetes Care 45(1):142–150 34782353 10.2337/dc21-1381 PMC8753768

[18] American Diabetes Association Professional Practice C (2024) 6. Glycemic goals and hypoglycemia: standards of care in Diabetes-2024. Diabetes Care 47(Suppl 1):S111–S25 38078586 10.2337/dc24-S006 PMC10725808

[19] Griffin WF, Choi AD, Riess JS, Marques H, Chang HJ, Choi JH et al (2023) AI evaluation of stenosis on coronary CTA, comparison with quantitative coronary angiography and fractional flow reserve: a CREDENCE trial substudy. JACC Cardiovasc Imaging 16(2):193–205 35183478 10.1016/j.jcmg.2021.10.020

[20] Lipkin I, Telluri A, Kim Y, Sidahmed A, Krepp JM, Choi BG et al (2022) Coronary CTA with AI-QCT interpretation: comparison with myocardial perfusion imaging for detection of obstructive stenosis using invasive angiography as reference standard. AJR Am J Roentgenol 219(3):407–19 35441530 10.2214/AJR.21.27289

[21] Choi AD, Marques H, Kumar V, Griffin WF, Rahban H, Karlsberg RP et al (2021) CT ​evaluation ​by ​artificial ​intelligence ​for ​atherosclerosis, stenosis and vascular ​morphology ​(CLARIFY): ​a ​multi-center, international study. J Cardiovasc Comput Tomogr 15(6):470–6 34127407 10.1016/j.jcct.2021.05.004

[22] Nurmohamed NS, Bom MJ, Jukema RA, de Groot RJ, Driessen RS, van Diemen PA et al (2024) AI-guided quantitative plaque staging predicts long-term cardiovascular outcomes in patients at risk for atherosclerotic CVD. JACC Cardiovasc Imaging 17(3):269–80 37480907 10.1016/j.jcmg.2023.05.020

[23] Nurmohamed NS, Gaillard EL, Malkasian S, de Groot RJ, Ibrahim S, Bom MJ et al (2024) Lipoprotein(a) and long-term plaque progression, low-density plaque, and pericoronary inflammation. JAMA Cardiol. 10.1001/jamacardio.2024.1874 39018040 10.1001/jamacardio.2024.1874 PMC11255968

[24] Nurmohamed NS, Min JK, Anthopolos R, Reynolds HR, Earls JP, Crabtree T et al (2024) Atherosclerosis quantification and cardiovascular risk: the ISCHEMIA trial. Eur Heart J. 10.1093/eurheartj/ehae471 39101625 10.1093/eurheartj/ehae471 PMC11439108

[25] Nurmohamed NS, Cole JH, Budoff MJ, Karlsberg RP, Gupta H, Sullenberger LE et al (2024) Impact of atherosclerosis imaging-quantitative computed tomography on diagnostic certainty, downstream testing, coronary revascularization, and medical therapy: the CERTAIN study. Eur Heart J Cardiovasc Imaging 25(6):857–66 38270472 10.1093/ehjci/jeae029 PMC11139521

[26] Shaw LJ, Blankstein R, Bax JJ, Ferencik M, Bittencourt MS, Min JK et al (2021) Society of Cardiovascular Computed Tomography / North American Society of Cardiovascular Imaging - expert consensus document on coronary CT imaging of atherosclerotic plaque. J Cardiovasc Comput Tomogr 15(2):93–109 33303383 10.1016/j.jcct.2020.11.002

[27] Lehto S, Ronnemaa T, Pyorala K, Laakso M (1999) Poor glycemic control predicts coronary heart disease events in patients with type 1 diabetes without nephropathy. Arterioscler Thromb Vasc Biol 19(4):1014–1019 10195930 10.1161/01.atv.19.4.1014

[28] Snell-Bergeon JK, Roman R, Rodbard D, Garg S, Maahs DM, Schauer IE et al (2010) Glycaemic variability is associated with coronary artery calcium in men with type 1 diabetes: the coronary artery calcification in type 1 diabetes study. Diabet Med 27(12):1436–42 21059097 10.1111/j.1464-5491.2010.03127.x PMC3052953

[29] Flynn MC, Kraakman MJ, Tikellis C, Lee MKS, Hanssen NMJ, Kammoun HL et al (2020) Transient intermittent hyperglycemia accelerates atherosclerosis by promoting myelopoiesis. Circ Res 127(7):877–92 32564710 10.1161/CIRCRESAHA.120.316653 PMC7486277

[30] Rawshani A, Rawshani A, Franzen S, Eliasson B, Svensson AM, Miftaraj M et al (2017) Range of risk factor levels: Control, Mortality, and cardiovascular outcomes in type 1 diabetes mellitus. Circulation 135(16):1522–1531 28416524 10.1161/CIRCULATIONAHA.116.025961 PMC5400410

