# Supplementary material for: Coronary plaque burden assessed by coronary CT angiography in individuals with type 1 diabetes and healthy controls
Source: Int J Cardiovasc Imaging. 2026 Feb 13;42(5):927–37. doi: 10.1007/s10554-026-03640-w (PMC13136224; doi:10.1007/s10554-026-03640-w)
Supplement: Supplementary file 1 — Supplementary Material 1 [file 10554_2026_3640_MOESM1_ESM.docx]

# **Supplementary material**

**Supplementary Table S1.** Plaque characteristics (**A**) and plaque volumes (**B**) type 1 diabetes participants vs. controls, adjusted for age, sex, systolic blood pressure, heart rate, body-mass index, LDL-C, triglycerides, and smoking history.

**A**

| **Characteristic** | **OR** | **p-value** |
| --- | --- | --- |
| Significant presence of coronary plaque | 2.71 (95% CI 0.9 to 9.07) | 0.087 |
| Presence of high-risk plaque | 4.86 (95% CI 0.79 to 53.98) | 0.124 |
| Presence of low-density plaque | 2 (95% CI 0.49 to 9.45) | 0.345 |
| Positive remodeling | 1.07 (95% CI 0.4 to 2.92) | 0.886 |

**B**

| **Characteristic** | **β-coefficient** | **p-value** |
| --- | --- | --- |
| Total plaque volume | 0.18 (95% CI -1.81 to 2.16) | 0.863 |
| Percent atheroma volume | 0.34 (95% CI -0.54 to 1.21) | 0.455 |
| Percent non-calcified plaque | 0.36 (95% CI -0.47 to 1.2) | 0.397 |
| Percent calcified plaque | 0.49 (95% CI -0.41 to 1.39) | 0.286 |

**Supplementary Table S2.** Characteristics of the individuals above median TIR versus below median TIR.

| **Characteristic** | **Above median TIR**  N = 24 | **Below median TIR**  N = 24 | **p-value** |
| --- | --- | --- | --- |
| Age ± SD – years | 41.2±5.0 | 43.3±5.3 | 0.147 |
| Male sex – no. (%) | 8 (33%) | 7 (29%) | 0.755 |
| Body mass index ± SD – kg/m^2^ | 26.1±5.4 | 25.4±3.7 | 0.926 |
| Active or previous smoking – no. (%) | 0.81±0.08 | 0.84±0.10 | 0.470 |
| Age ± SD – years | 9 (38%) | 9 (38%) | 1.000 |
| Systolic blood pressure ± SD – mmHg | 125±12 | 122±17 | 0.236 |
| Diastolic blood pressure ± SD – mmHg | 78±7 | 76±10 | 0.236 |
| ACE inhibitor or ARB use – no. (%) | 0 (0%) | 2 (8.3%) | 0.489 |
| LDL cholesterol ± SD – mmol/L | 2.58±0.90 | 2.57±0.93 | 0.877 |
| HDL cholesterol ± SD – mmol/L | 1.79±0.69 | 1.95±0.41 | 0.035 |
| Triglycerides (IQR) – mmol/L | 0.78 (0.61, 1.25) | 0.67 (0.56, 1.01) | 0.427 |
| Lipoprotein(a) (IQR) – nmol/L | 15 (7, 18) | 20 (11, 122) | 0.053 |
| C-reactive protein (IQR) – mg/L | 0.90 (0.60, 2.03) | 0.85 (0.60, 1.93) | 0.760 |
| Age of diabetes onset (IQR) – years | 14 (9, 20) | 20 (14, 28) | 0.062 |
| Disease duration (IQR) – years | 27 (20, 32) | 23 (16, 29) | 0.243 |
| Diabetic retinopathy – no. (%) | 6 (25%) | 9 (38%) | 0.350 |
| Proliferative retinopathy – no. (%) | 3 | 0 | 0.234 |
| Diabetic neuropathy – no. (%) | 0 (0%) | 2 (8.3%) | 0.489 |
| Urine microalbumin-creatinine ratio (IQR) – mg/mmol | 0.37 (0.20, 0.57) | 0.28 (0.00, 0.47) | 0.143 |
| HbA1c ± SD – mmol/mol | 48±6 | 58±9 | 0.000 |
| Cumulative HbA1c exposure ± SD – mmol/mol * years | 1,263±569 | 1,357±609 | 0.631 |
| Insulin dose ± SD – units/day | 42±18 | 45±18 | 0.621 |
| CGM sensor type – no. (%) |  |  | 0.003 |
| Freestyle Libre 2 | 4 (17%) | 16 (67%) |  |
| Freestyle Libre 3 | 2 (8.3%) | 1 (4.2%) |  |
| Medtronic Guardian | 9 (38%) | 3 (13%) |  |
| Dexcom G6 | 9 (38%) | 4 (17%) |  |
| Days of CGM data (IQR) | 754 (596, 810) | 532 (484, 684) |  |
| TIR (IQR) – % | 79 (75, 87) | 54 (43, 67) | 0.000 |
| TAR (IQR) – % | 17 (9, 21) | 42 (28, 53) | 0.000 |
| TBR (IQR) – % | 2.1 (1.1, 4.9) | 2.6 (1.0, 7.6) | 0.657 |
| Glucose CV ± SD – % | 31±8 | 38±5 | 0.000 |
| Hypoglycemic events (IQR) – no. per 90 days | 33 (21, 78) | 41 (13, 74) | 0.767 |
| Insulin pump no. (%) |  |  | 0.004 |
| None | 5 (21%) | 12 (50%) |  |
| Manual | 4 (17%) | 8 (33%) |  |
| Predictive low-glucose suspend | 0 (0%) | 1 (4.2%) |  |
| Hybrid cosed-loop | 11 (46%) | 3 (13%) |  |
| DIY closed-loop | 4 (17%) | 0 (0%) |  |

ACE, angiotensin-converting enzyme; ARB, angiotensin receptor blocker; LDL, low-density lipoprotein; HDL, high-density lipoprotein; TIR, time in rage; TAR, time above range; TBR, time below range; glucose CV, glucose coefficient of variation.

**Supplementary Table S3.** Plaque burden of the individuals above median TIR versus below median TIR. Unadjusted associations (**A**), and adjusted for age, sex, systolic blood pressure, body-mass index, LDL-C and smoking history (**B** and **C**).

**A**

| **Characteristic** | **Above median TIR**  N = 24 | **Below median TIR**  N = 24 | **p-value** |
| --- | --- | --- | --- |
| Significant presence of coronary plaque – no. (%) | 6 (25%) | 8 (33%) | 0.525 |
| Total plaque volume (IQR) – mm^3^ | 6 (2, 14) \| 20±43 | 6 (0, 12) \| 22±49 | 0.552 |
| Percent atheroma volume (IQR) – % | 0.20 (0.08, 0.46) | 0.22 (0.00, 0.57) | 0.594 |
| Percent non-calcified plaque volume (IQR) – % | 0.19 (0.07, 0.43) | 0.22 (0.00, 0.57) | 0.594 |
| Percent calcified plaque volume (IQR) – % | 0.01 (0.00, 0.03) | 0.00 (0.00, 0.03) | 0.568 |
| Low-density plaque volume (IQR) – % | 0.000 (0.000, 0.000) | 0.000 (0.000, 0.000) | 0.546 |
| Presence of high-risk plaque – no. (%) | 5 (21%) | 3 (13%) | 0.701 |
| Presence of low-density plaque – no. (%) | 5 (21%) | 3 (13%) | 0.701 |
| Positive remodeling – no. (%) | 7 (29%) | 9 (38%) | 0.540 |

**B**

| **Characteristic** | **OR** | **p-value** |
| --- | --- | --- |
| Significant presence of coronary plaque | 0.81 (95% CI 0.15 to 4.39) | 0.797 |
| Presence of high-risk plaque | 9.59 (95% CI 0.8 to 375.63) | 0.131 |
| Presence of low-density plaque | 9.59 (95% CI 0.8 to 375.63) | 0.131 |
| Positive remodeling | 0.73 (95% CI 0.18 to 2.92) | 0.654 |

**C**

| **Characteristic** | **β-coefficient** | **p-value** |
| --- | --- | --- |
| Total plaque volume | 2.11 (95% CI -0.48 to 4.71) | 0.118 |
| Percent atheroma volume | 0.53 (95% CI -0.68 to 1.73) | 0.397 |
| Percent non-calcified plaque | 0.51 (95% CI -0.65 to 1.66) | 0.397 |
| Percent calcified plaque | 0.09 (95% CI -1.01 to 1.19) | 0.873 |

**Supplementary Figure S1.** Associations between HbA1c (mmol/mol) and coronary plaque burden in the CGM cohort.


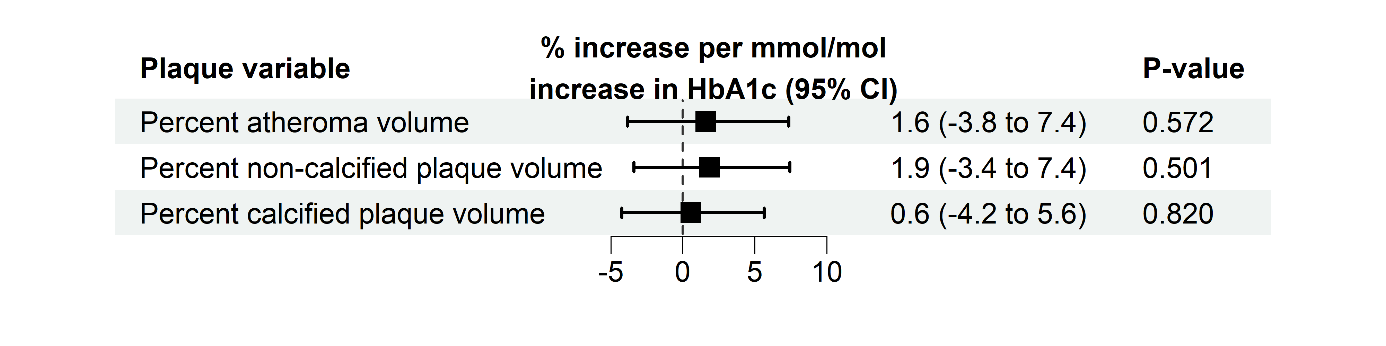
 Shown are the relative increases derived beta coefficients from linear regression model of HbA1c with the different plaque volumes (log_2_ transformed) as outcome variable. Models were adjusted for clinical risk factors (age, sex, systolic blood pressure, body mass index, LDL-C, triglycerides, smoking history, disease duration, presence of diabetic retinopathy, and family history of CVD).

**Supplementary Figure S2.** Associations between age at onset of diabetes and coronary plaque burden in the CGM cohort.


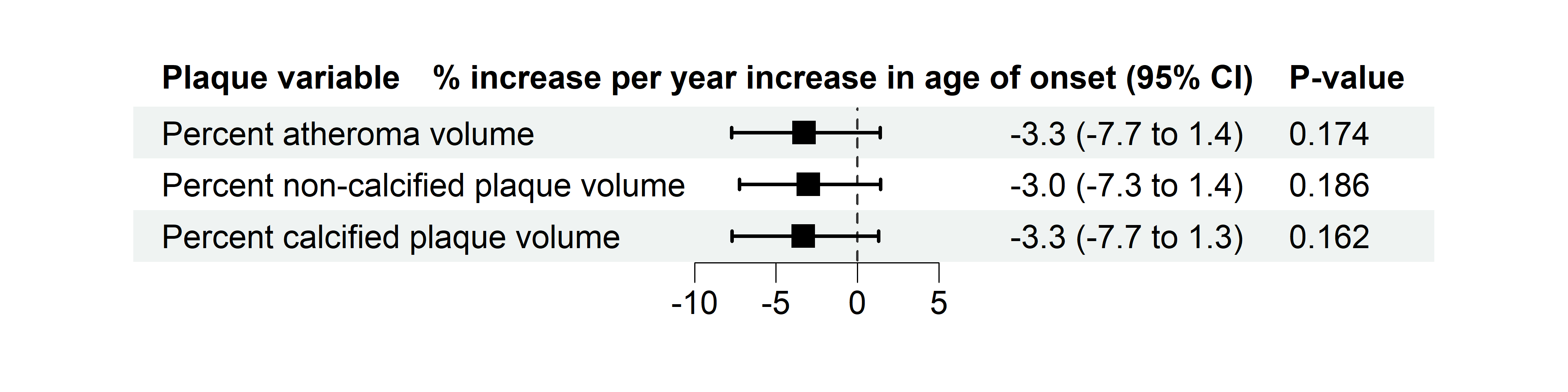


Shown are the relative increases derived beta coefficients from linear regression model of age at onset with the different plaque volumes (log_2_ transformed) as outcome variable. Models were adjusted for age.

**Supplementary Figure S3**. Associations between LDL cholesterol and coronary plaque burden in all participants with diabetes (**A**), the healthy control participants (**B**), and all participants (**C**).


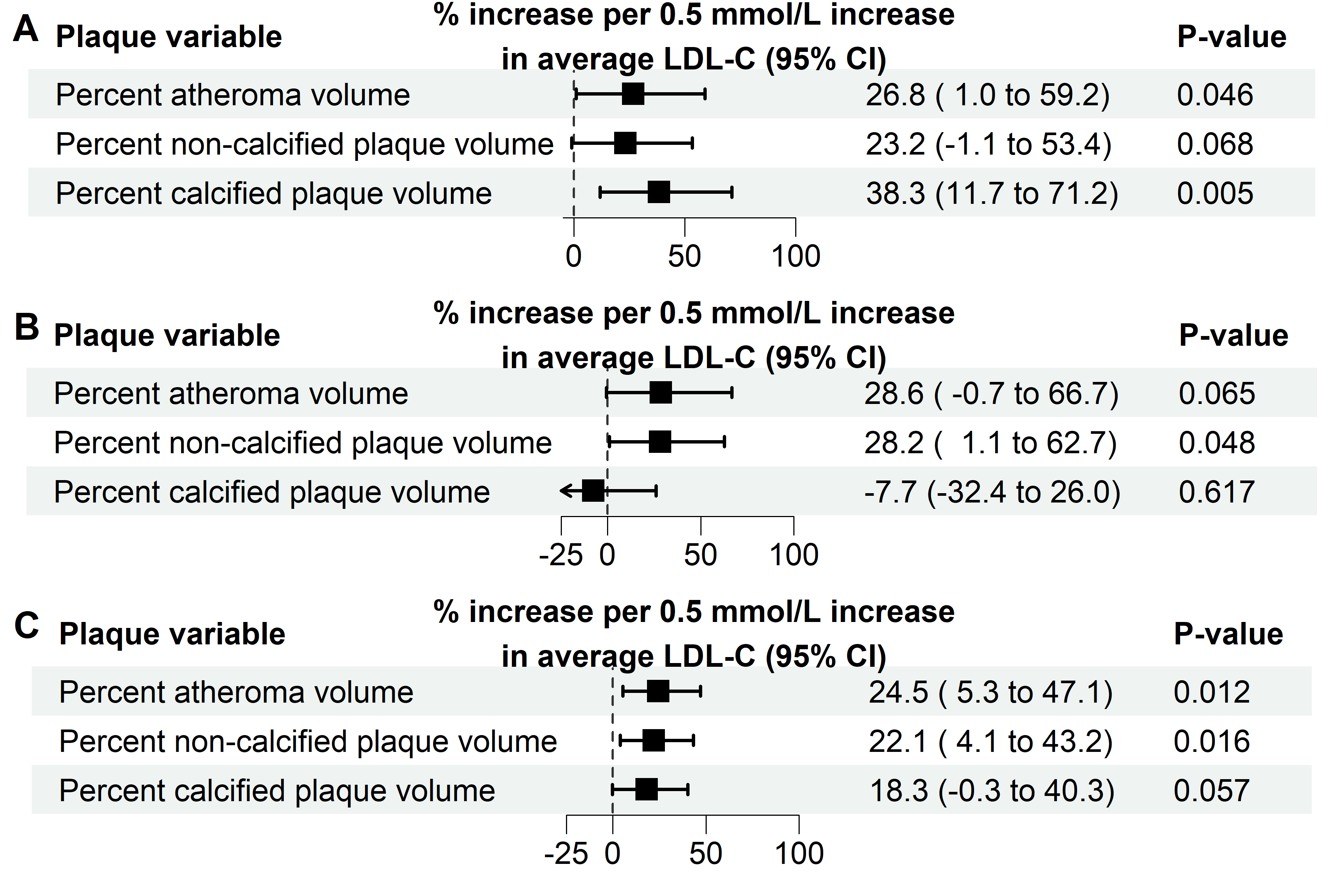


Shown are the relative increases derived beta coefficients from linear regression model of LDL-cholesterol with the different plaque volumes (log_2_ transformed) as outcome variable. Adjusted for sex, age, BMI, systolic blood pressure, history of smoking, family history of CVD, triglycerides and heart rate. LDL-C, low-density lipoprotein cholesterol.

No interaction was observed between groups (type 1 diabetes, healthy controls) for percent atheroma volume (p = 0.442) and percent non-calcified plaque volume (p = 0.544), for percent calcified plaque volume, an interaction was observed (p = 0.025).
